# Rapid-developed primary malignant myoepithelioma in the cavernous sinus: a case report

**DOI:** 10.1186/1471-2377-13-40

**Published:** 2013-05-04

**Authors:** Yuan Hong, Song-Xue Guo, Sheng Chen, Damon Klebe, Jian-Min Zhang, Qun Wu

**Affiliations:** 1 Department of Neurosurgery, 2nd Affiliated Hospital, School of Medicine, Zhejiang University, 88# Jiefang Road, Hangzhou, Zhejiang 310009, China; 2 Department of Neurosurgery, Sir Run Run Shaw Hospital, School of Medicine, Zhejiang University, 3# East Qingchun Road, Hangzhou, Zhejiang 310016, China; 3 Department of physiology, Loma linda university, Risley Hall 11041 Campus Street, Loma Linda, CA 92350, USA

**Keywords:** Myoepithelioma, Cavernous sinus, Treatment, Pathology

## Abstract

**Background:**

Malignant myoepithelioma is a relatively rare malignant tumor occurring most frequently in the salivary glands. A few isolated cases have been described in other locations, including soft tissue, bone, lung, bronchus, oral cavity, nasopharynx, larynx, and maxillary sinus. Malignant myoepithelioma, however, is uncommonly involved within the cavernous sinus. To the best of our knowledge, this is the first report of malignant myoepithelioma arising from within the cavernous sinus.

**Case presentation:**

Herein, we report a case of a 48-year-old woman who presented a 1-month history of diplopia and blepharoptosis as well as radiological evidence of a rapidly developing cavernous sinus tumor. The patient underwent a trans-sphenoidal biopsy and a histological diagnosis indicated a malignant myoepithelioma. After diagnosis, the tumor grew rapidly and her clinical condition deteriorated progressively. Therefore, a pterional craniotomy with partial tumor removal was performed. The patient’s clinical state was worsened, and she died two months after the initial operation. Because the malignant myoepithelioma could not be traced to an organ of origin, other than the cavernous sinus, this case was diagnosed as a primary intracranial malignant myoepithelioma.

**Conclusion:**

The purpose of presenting this case report is to raise awareness among clinicians to consider malignant myoepithelioma as a differential diagnosis when a cavernous sinus mass is identified. Furthermore, an ideal management strategy for malignant myoepithelioma is not known and the prognosis seems to be unfavorable; therefore, more cases are needed to enhance our knowledge of the diagnosis, treatment, and prognosis of this rare intracranial lesion.

## Background

Malignant myoepithelioma, also called myoepithelial carcinoma, is a malignant tumor that usually occurs in the salivary glands. It contains markedly proliferating myoepithelial cells, which are normally present in the salivary, mammary, and sweat glands [1,2]. Less commonly, these tumors have been found in the soft tissue [3], bone [4], lung [5], bronchus [6], oral cavity [7,8], nasopharynx [9], larynx [10], and maxillary sinus [11]. However, primary intracranial myoepithelioma is extremely rare, and only two cases have been described according to our literature search. Neither of these reported cases involved the cavernous sinus. Here, we report a rare case of malignant myoepithelioma in the cavernous sinus and discuss its clinical, histopathological, and immunohistochemical features, as well as its prognosis.

## Case presentation

**Figure 1:**
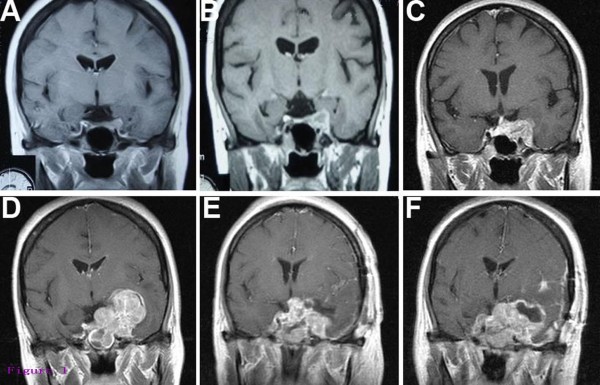
**Coronal MRI scans of malignant myoepithelioma development in the present case.** Initial T1-weighted post-contrast images taken at symptom onset were normal (**A**). Follow-up images 1 month later depicted a new, left cavernous sinus mass with heterogeneous signal intensity (**B**). Preoperative images indicated the tumor mass significantly increased (**C**). Post-biopsy images displayed more progressive tumor development, since the tumor filled the left cavernous sinus and extended into the ipsilateral middle fossa (**D**). Postoperative images presented a large residue with successive tumor growth (**E, F**).

A 48-year-old woman was admitted to our hospital with symptoms of diplopia and blepharoptosis for one month. Before being taken to our hospital, the patient had visited the neurology departments in other hospitals with the aforementioned symptoms and subsequent Magnetic Resonance Imaging (MRI) (Figure 1A) and endocrine assessments did not indicate anything abnormal. Although corticosteroid therapy ameliorated her symptoms, these symptoms reappeared after drug withdrawal. One month later, a repeated MRI presented a mass lesion located in her left cavernous sinus with an obscure boundary. The lesion was isointense in T1-weighted MRI sequences and hypointense in T2-weighted sequences. After intravenous contrast agent administration, T1-weighted images showed intense and inhomogeneous enhancement of the mass (Figure 1B). She was referred to our hospital for further evaluation and treatment.

At the time of admission, physical examination of the patient showed abnormal function in her left eye such as blepharoptosis, a 4 mm-diameter pupil with light reflex retardation, limited movement, and visual field loss of upside, downside, and temporal side, which were considered to be consequences of homo-side oculomotor and abducent nerves paralysis caused by mass pressure. Signs of reduction in pain and touch sensation on the left forehead skin were also observed in the physical examination. No abnormal function from other cranial nerves was observed. The pituitary hormones test showed nothing unusual. A general examination, including chest CT scan, abdominal ultrasound exam, bone X-ray, and nasal endoscopic check did not reveal any primary or metastatic lesions in other areas.

**Figure 2:**
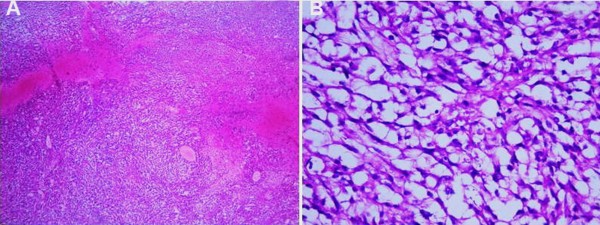
**Histological photomicrographs.** Hematoxylin and eosin stain (×50) shows an epithelioid neoplasm composed of hyper-cellular, moderately pleomorphic round to polygonal tumor cells embedded in a myxoid stroma. There are areas of hemorrhage or necrosis (**A**). Hematoxylin and eosin stain (× 400) Higher power magnification demonstrates the cells have round to ovoid nuclei, prominent nucleoli, and abundant eosinophilic cytoplasm. Mitotic figures are present (**B**).

**Figure 3:**
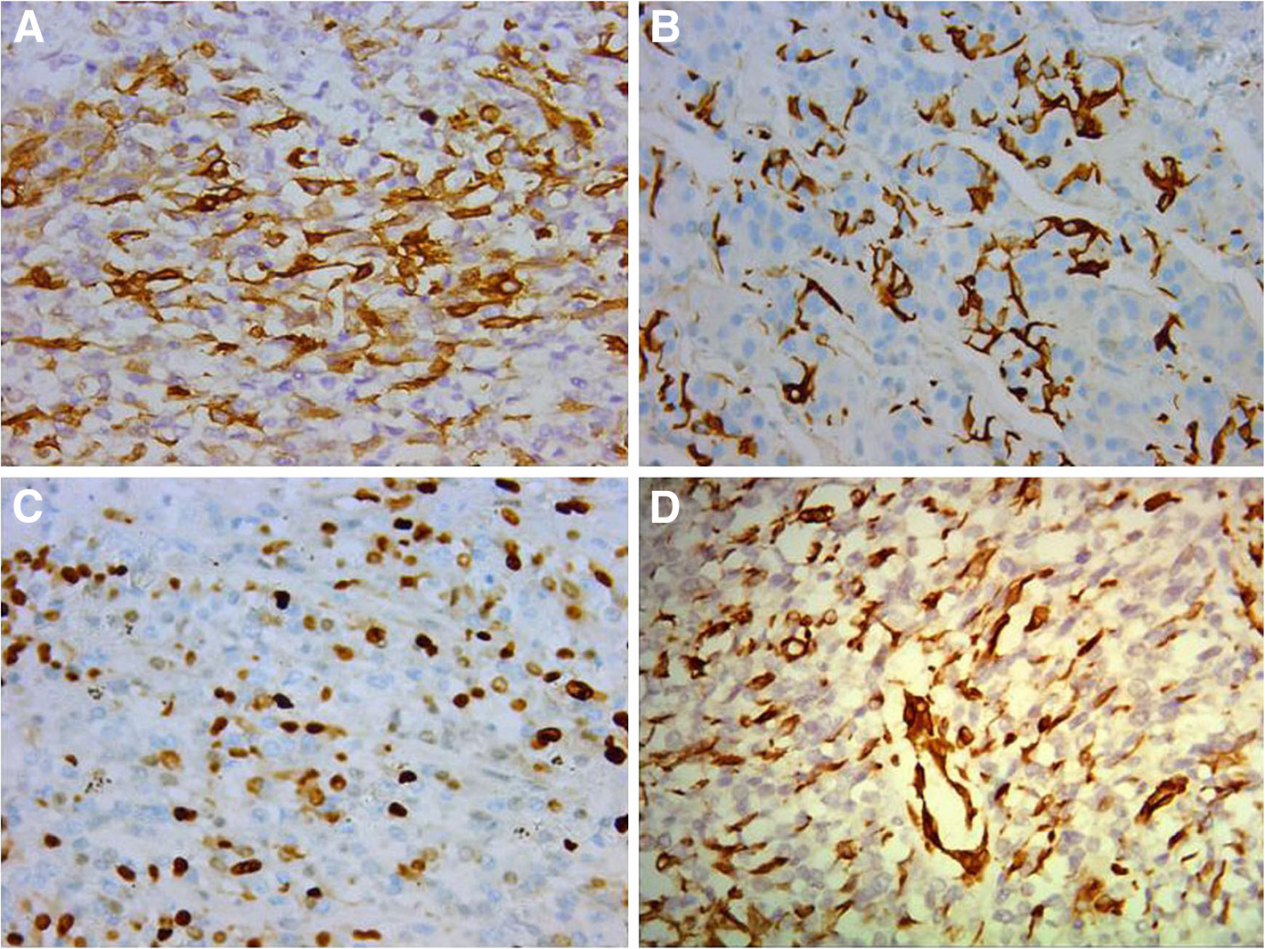
**Immunohistochemical stains.** The tumor cells demonstrated immunoreactivity for SMA (**A**), GFAP (**B**), S-100 (**C**), vimentin (**D**). Original magnification × 400.

The preoperative differential diagnosis included pituitary adenoma invading the cavernous sinus, granulomatous inflammation, metastatic brain tumor, and primary malignant tumor arising in the cavernous sinus. Therapeutic trials of corticosteroid administration are important diagnostic tests to differentiate granulomatous inflammation from other neoplastic lesions in the cavernous sinus [12]. As aforementioned, the clinical symptoms of the patient were partially ameliorated after corticosteroid treatment in the other hospitals, thus we used corticosteroid therapy again on her for assisting diagnosis. Corticosteroid therapy relieved her blepharoptosis, but it did not ameliorate her other symptoms, such as reduced sensation and pupil function. Furthermore, the patient felt more severe headaches, and the results from another MRI scan indicated a larger scope of mass than before (Figure 1C). With consideration to a malignant tumor and for making an accurate diagnosis, we recommended performing an endoscopic trans-sphenoidal approach biopsy, and the patient and her family agreed with our suggestion. The biopsy was performed, and digital pathological specimens of this rubbery mass were sent to the Department of Pathology and Laboratory Medicine at the UCLA Medical Center. The returned surgical pathology consult report made the final diagnosis of malignant myoepithelioma. Histological investigation of specimens revealed a tumor composed of hyper-cellular, moderately pleomorphic round to polygonal tumor cells with moderate to marked nuclear atypia and eosinophilic cytoplasm. Mitotic figures were present. There are areas of hemorrhage and necrosis (Figure 2). Immunohistochemistry (IHC) demonstrated the tumor cells were positive for smooth muscle actin (SMA), glial fibrillary acidic protein (GFAP), S-100, and vimentin (Figure 3). The MIB-1 (Ki-67) proliferation index of rhabdoid and spindle-shaped cells were 60%. Staining for Desmin, EMA, CK5/6, CD138, myosin, HMB45, CD79a, and CD45 was negative. The histological findings of the tumor were compatible with malignant myoepithelioma.

The patient was then recommended for postoperative radiotherapy and chemotherapy. Due to economic reasons, they refused the adjuvant treatment. After biopsy, her left pupil progressively enlarged to the state of mydriasis, while the diameter of her right pupil also gradually increased to 5 mm with a reduction in light reflex. Physical examination revealed typical symptoms resulting from impacted trigeminal and hypoglossal nerves, including reduced facial pain and touch sensation, diminished corneal reflex, dysphagia, cough, and hoarseness. The general condition of the patient progressively deteriorated. A repeated MRI showed more severe tumor development, which filled the left cavernous sinus and extended into the ipsilateral middle fossa (Figure 1D). Given the lesion’s expansion and the patient’s clinical deterioration, a pterional craniotomy for resection of the progressed tumor to relieve intracranial pressure of the patient was performed. Only incomplete surgical resection was accomplished because the tumor extended into the hypothalamus and enclosed the left cavernous carotid artery. Postoperative MRI presented a large residue and successive tumor growth (Figure 1E, F). This pathological finding was compatible with the initial biopsy finding,and the final pathological diagnosis was considered as malignant myoepithelioma of the cavernous sinus. The patient died two weeks after the second operation due to rapid clinical deterioration, approximately four months after onset of the initial symptoms. Autopsy was not performed.

## Discussion

Malignant myoepithelioma is a rare malignant neoplasm in which the tumor cells show myoepithelial differentiation. They are most often found in salivary glands but also occur in other tissues and organs, including soft tissues where they were recently recognized as a distinct diagnostic entity [13]. Malignant myoepitheliomas of soft tissue display the same wide range of histologic features as those of salivary gland origin. It appears that these neoplasms are equally common in males and females and occur in a wide age range with a peak in the third to fifth decades [14]. Unlike its salivary counterpart, a larger percentage of soft tissue myoepithelial tumors demonstrate a more aggressive histology and show a greater propensity for metastatic disease [15]. Furthermore, they are distinguished from benign myoepitheliomas on the basis of their cytologic rather than architectural features. They are infrequently associated with a preexisting benign tumor but appear to arise de novo [14].

Preoperative diagnosis may be difficult or impossible for malignant myoepithelioma due to nonspecific clinical manifestation and imaging characteristics. Histological examination supplemented with IHC staining studies, is the most reliable and conclusive method of diagnosis. From a pathological point-of-view, malignant myoepithelioma commonly contains marked proliferating myoepithelial cells, and various morphologically, neoplastic myoepithelial cells are presented, including spindle, plasmacytoid, clear, epithelioid and stellate types [16,17]. The presence of significant atypia, atypical mitotic figure, hemorrhage, and necrosis has been considered features of malignancy [14]. An immunohistochemical study using a panel of epithelial and myogenic markers is essential for diagnosing this tumor. Myoepithelial tumors are characteristically positive for myoepithelial markers including vimentin, cytokeratin, SMA, S100 and GFAP [2,14,18].

In this case, the tumor commonly consists of spindle cells with moderate to marked nuclear atypia. The tumor malignancy is supported by cellular pleomorphism, nuclear atypia, hemorrhage, necrotic areas, a mitotic activity and a high proliferative MIB-1 index in the neoplasm under the light microscope. There are some tumors that also show similar spindle cell composition, such as parachordoma, chordoma, extraskeletal myxoid chondrosarcoma, unusual carcinoma and even chondrosarcoma if a chondroid matrix is present and the sampling is small. Distinguishing malignant myoepithelioma from other tumors is as difficult radiographically as it is clinically. Therefore, to make the final diagnosis in the present case, immunohistochemical studies were necessary. The expression patterns of the epithelial and myogenic markers are somewhat varied in each case and may reflect the degree of myoepithelial cell differentiation. The combination of SMA, GFAP, S-100, and vimentin positive cells in these lesions aids in the diagnosis of malignant myoepithelioma.

Intracranial primary malignant myoepithelioma is extremely rare and has never been noted within the cavernous sinus. In view of this rare site for malignant myoepithelioma, we only found two cases reporting intracranial primary malignant myoepithelioma utilizing the help of Medline and Pubmed. Although some differences exist between the three cases, we can still explore possible explanations for the occurrence of this salivary gland tumor. Carsten Nieder et al. [19] had reported a similar case with a primary myoepithelial carcinoma in the sellar region. The tumor in this case showed a longer developmental progression with no invasion into the cavernous sinus. In addition, Sibel Erdogan et al. [20] described a case of myoepithelial carcinoma arising in the intracranial dura outside of the sellar region. In comparison to these two cases, our case showed a rapid tumor development (the patient survived only four months from initial onset of symptoms to death) and a rare invasion into the cavernous sinus.

The origin of the malignant myoepithelioma in the present case remains obscure. At first, we tried to find some evidence of an extracranial source with salivary gland distribution to explain it as a metastatic tumor. However, with regard to the negative findings in the chest CT scan and nasal endoscopic check, we excluded possible primary sources, such as the bronchus, nasopharynx, etc. Therefore, the primary tumor source in this case is determined to be the cavernous sinus, while taking into consideration the occurrence of salivary gland heterotopia. Histologically, the sellar region is a neighboring site to the oral cavity, nasopharynx, and larynx, where there are salivary glands existing. And some known reports presented some sellar region salivary gland tumors that may be related to salivary gland rests in the sellar region or pituitary [21,22]. In addition, a possible origin of multipotential stem cells also needs to be taken into consideration.

Since intracranial malignant myoepitheliomas are such a rare entity, effective management strategies have not been established. Treatment options include surgical resection, chemotherapy, radiotherapy, or a combination of these approaches. Surgical resection is the mainstay therapy for malignant myoepithelioma, but in some circumstances it may not be technically feasible due to the rich vascularity and involvement of the cavernous sinus and carotid arteries. Radiation therapy and chemotherapy can also be used for malignant myoepithelioma. However, there is no consensus whether postoperative adjuvant therapy is even required because the prognosis of this condition is unclear. Some authors reported radiotherapy or concurrent chemoradiotherapy is effective for local recurrence and distant metastasis [23,24]. But other reports confirmed the lack of effectiveness of this adjuvant treatment for both local and distant recurrences [19,25]. The overall prognosis of malignant myoepithelioma is poor. Several studies reported aggressive clinical behaviors for malignant myoepithelioma. The average metastatic rate was 47% and the mortality rate was 29% after a mean of 32 months [24]. Some factors have been evaluated as potential prognostic indicators, including clinical stage, site and size of the tumor, high proliferative activity, extensive invasion into the surrounding tissue, perineural permeation, the abnormal presence of nuclear DNA content, and marked cellular pleomorphism [26]. In our case, the course of the disease was very unfavorable because complete tumor resection could not be achieved, radiotherapy or chemotherapy had not been used, high proliferative activity, and other factors.

## Conclusion

In conclusion, intracranial malignant myoepithelioma is an extremely rare or often misdiagnosed lesion. This is the first reported case of a malignant myoepithelioma arising within the cavernous sinus. Preoperative diagnosis may be difficult or impossible due to nonspecific clinical manifestation and imaging characteristics, thus diagnosis can only be made postoperatively based on histopathological and immunohistochemical analysis. The purpose of presenting this case report is to raise awareness among clinicians to consider this clinical entity as a differential diagnosis when a cavernous sinus mass is identified. Furthermore, ideal management strategy is not known. Surgical resection has been the accepted treatment for malignant myoepithelioma. The role of chemotherapy or radiotherapy, however, is controversial. The prognosis of malignant myoepithelioma seems to be unfavorable; therefore, more cases are needed to enhance our knowledge of the diagnosis, treatment, and prognosis of this rare intracranial lesion.

## Consent

Written informed consent was obtained from the patient for publication of this case report and any accompanying images. A copy of the written consent is available for review by the Editor-in-Chief of this journal.

## Abbreviations

MRI: Magnetic Resonance Imaging; IHC: Immunohistochemistry; SMA: Smooth muscle actin; GFAP: Glial fibrillary acidic protein.

## Competing interests

All authors declare no competing interest.

## Authors’ contributions

Hong drafted the first manuscript and made a contribution to acquisition and interpretation of data. Chen, Guo and Zhang performed the clinical work-up and literature search. Klebe revised the language and grammar of the manuscript. Hong and Wu revised the manuscript that led to the final approval of the current submission. All authors read and approved the final manuscript.

## Pre-publication history

The pre-publication history for this paper can be accessed here:

http://www.biomedcentral.com/1471-2377/13/40/prepub
